# Arsenic Exposure Is Associated with Decreased DNA Repair *in Vitro* and in Individuals Exposed to Drinking Water Arsenic

**DOI:** 10.1289/ehp.9008

**Published:** 2006-05-10

**Authors:** Angeline S. Andrew, Jefferey L. Burgess, Maria M. Meza, Eugene Demidenko, Mary G. Waugh, Joshua W. Hamilton, Margaret R. Karagas

**Affiliations:** 1 Department of Community and Family Medicine, Section of Biostatistics and Epidemiology, Dartmouth Medical School, Lebanon, New Hampshire, USA; 2 Department of Environmental and Community Health, University of Arizona, Tucson, Arizona, USA; 3 Department of Natural Resources, Technological Institute of Sonora (ITSON), Ciudad Obregon, Sonora, Mexico; 4 Department of Pharmacology and Toxicology, Dartmouth Medical School, Hanover, New Hampshire, USA

**Keywords:** arsenic, arsenite, DNA repair, ERCC1, molecular epidemiology, nucleotide excision repair

## Abstract

The mechanism(s) by which arsenic exposure contributes to human cancer risk is unknown; however, several indirect cocarcinogenesis mechanisms have been proposed. Many studies support the role of As in altering one or more DNA repair processes. In the present study we used individual-level exposure data and biologic samples to investigate the effects of As exposure on nucleotide excision repair in two study populations, focusing on the excision repair cross-complement 1 (ERCC1) component. We measured drinking water, urinary, or toenail As levels and obtained cryopreserved lymphocytes of a subset of individuals enrolled in epidemiologic studies in New Hampshire (USA) and Sonora (Mexico). Additionally, in corroborative laboratory studies, we examined the effects of As on DNA repair in a cultured human cell model. Arsenic exposure was associated with decreased expression of ERCC1 in isolated lymphocytes at the mRNA and protein levels. In addition, lymphocytes from As-exposed individuals showed higher levels of DNA damage, as measured by a comet assay, both at baseline and after a 2-acetoxyacetylaminofluorene (2-AAAF) challenge. In support of the *in vivo* data, As exposure decreased ERCC1 mRNA expression and enhanced levels of DNA damage after a 2-AAAF challenge in cell culture. These data provide further evidence to support the ability of As to inhibit the DNA repair machinery, which is likely to enhance the genotoxicity and mutagenicity of other directly genotoxic compounds, as part of a cocarcinogenic mechanism of action.

Arsenic is an established lung, skin, and bladder carcinogen [International Agency for Research on Cancer (IARC) 2004]; however, the carcinogenic mechanisms are currently under investigation. Based primarily on studies of highly exposed populations in Taiwan and elsewhere, the U.S. Environmental Protection Agency (EPA) recently reduced the maximum contaminant level (MCL) standard for arsenic in drinking water from 50 μg/L to 10 μg/L (U.S. EPA 2006). At the lower end of the dose–response curve, the biologic effects and magnitude of disease risk in the human population remain unknown (Abernathy et al. 1999). However, a growing number of laboratory studies, both in cell cultures and in experimental animals, have demonstrated biologic effects of As at very low levels equivalent to those below the new 10 μg/L standard. These effects include endocrine disruption, altered cell signaling, altered cell cycle kinetics, alterations in proliferative response, and other effects that may be associated with carcinogenesis and other disease processes (reviewed by Rossman 2003). Thus, it is important to understand the potential adverse effects of such exposure in the human population.

An estimated 2% of the drinking water serving U.S. households contains ≥ 2 μg/L As (National Research Council 2001). Approximately 40% of households in the state of New Hampshire are served by unregulated private wells, with homeowner-financed, optional contaminant testing. Moreover, until recent studies revealed the extent of geologic As contamination of drinking water in the state (Peters et al. 1999), As was not part of the standard laboratory water testing panel. Case–control studies of bladder and skin cancer conducted in the New Hampshire population have detected evidence of elevated cancer risks. For bladder cancer, an excess risk was observed primarily among smokers exposed to As in the drinking water, supporting hypotheses that these levels of As are cocarcinogenic (Karagas et al. 2001, 2004). Likewise, drinking water in the southwestern United States and northern Mexico contains As at concentrations above the new MCL of 10 μg/L (Meza et al. 2004).

The precise mechanism of As cocarcinogenesis is unknown. It has been difficult to detect genotoxic effects of As per se at environmental levels [Agency for Toxic Substances and Disease Registry (ATSDR) 1999; IARC 2004; Rossman 2003]. However, many studies support the role of As in altering one or more DNA repair processes [World Health Organization (WHO) 2001]. Arsenic has been shown to potentiate the genotoxicity of other organic mutagen-carcinogens, particularly poly-cyclic aromatic hydrocarbons (PAHs), including benzo[*a*]pyrene (BAP) and ultraviolet radiation (UVR) (ATSDR 1999; Rossman 2003). Rats treated with As and BAP sustained adduct burdens longer than did rats treated with BAP alone, suggesting impairment of DNA repair by As as a possible mechanism (Tran et al. 2002). A study using human fibroblasts found that low (2.5 μM, ~ 180 μg/L) concentrations of arsenite reduced nucleotide excision repair efficiency, and incision frequency in particular, after UVR exposure (Hartwig et al. 1997). Results of another study in cultured human fibroblasts indicated that As exposure reduced DNA repair capacity as measured by the comet assay (Curnow et al. 2001). The effects of As are strongly dose, time, and species dependent (Barchowsky et al. 1999; WHO 2001). In particular, several As-induced effects exhibit a biphasic characteristic. For example, low (≤ 1–2 μM) doses of As in cell culture increase cell proliferation and enhance endocrine signaling, whereas higher but still noncytotoxic doses (2–5 μM) suppress these same pathways (Barchowsky et al. 1999). Likewise, patterns of altered gene expression, as detected by DNA microarray studies, demonstrated very different patterns at low versus higher doses (Andrew et al. 2003b). Thus, although animal and cell culture studies provide controlled model systems for mechanistic studies of As, it is critical to understand which of these findings translate into cellular, molecular, and clinical effects in actual human exposure situations, and the role of these in pathophysiologic processes such as carcinogenesis. In our preliminary study of human lymphocytes from individuals exposed to drinking water As, As exposure was correlated in a strongly dose-dependent manner to decreased expression of three nucleotide excision repair genes: *ERCC1*, *XPB*, and *XPF* (Andrew et al. 2003a).

The objective of this investigation was to evaluate our preliminary observation of an association between As exposure, focusing on *ERCC1* gene expression levels in a larger number of individuals with exposure data and biologic samples. In addition to gene expression, we investigated the effects of As exposure at both the protein and DNA repair functional levels. We then extended our investigation into another population exposed to similar levels of As in Mexico and also performed *in vitro* As experiments to validate our results in a controlled system.

## Materials and Methods

### Human populations

#### New Hampshire

We selected subjects from an ongoing epidemiologic study of bladder cancer in New Hampshire (Karagas et al. 1998, 2004). Selection was based on high or low As exposure from individuals on whom we had collected cryopreserved lymphocytes. Within this subset (*n* = 37), the drinking water As levels of the low-exposure group averaged 0.7 μg/L (range, 0.007–5.3 μg/L), whereas the high-exposure group averaged 32 μg/L (range, 10.4–74.7 μg/L). Data on subject’s exposure history were available through a personal interview covering demographic information, history of tobacco use, and other lifestyle factors. Informed consent was obtained from each participant, and all procedures and study materials were approved by the Committee for the Protection of Human Subjects at Dartmouth College.

Subjects agreed to provide a venous blood sample that was drawn into cell prep tubes (CPT) containing citrate and a lymphocyte isolation gradient. Blood tubes were maintained at 4°C and sent to the study laboratory for processing and analysis. No later than 24 hr after the blood draw, the lymphocytes collected in CPTs containing sodium citrate were isolated according to the manufacturer’s instructions using standard buoyant density centrifugation methods. After centrifugation, first plasma was removed, aliquoted, and frozen at −80°C, and then the mononuclear cells were removed by pipette and cryo-preserved (−120°C) using freezing media at a controlled rate of 1°C/min. This procedure has previously been demonstrated to ensure approximately 90% viability after thawing (Wei et al. 1994).

Additionally, toenail clipping samples collected at the time of interview were analyzed for As and other trace elements by instrumental neutron activation analysis (INAA) at the University of Missouri research reactor using a standard comparison approach as described previously (Cheng et al. 1995). The detection limit for As measured by INAA is approximately 0.001 μg/g. A water sample from the current household drawn into commercially washed (mineral-free) high-density polyethylene bottles (Fisher Scientific, Suwanee, GA, USA) that meet U.S. EPA standards for water collection (U.S. EPA 2002) were analyzed for As concentration using an Agilent 7500c Octopole inductively coupled plasma mass spectrometer (Agilent Technologies Inc., Palo Alto, CA, USA) in the Dartmouth Trace Element Analysis Core Facility (Karagas et al. 2000).

#### Sonora, Mexico

Subjects were recruited in 2004 from several towns in the Yaqui Valley of Sonora, Mexico, by contact through local health care officials after attending an informational meeting in their hometowns, as described previously (Meza et al. 2004). The present study involved a subset of subjects who provided biologic samples (*n* = 16). They ranged in age from 23 to 63 years and were in good health (self-reported and by physical examination). All subjects gave their informed consent, as approved by the Human Subjects Committee of the University of Arizona and the Ministry of Public Health of Sonora State. Physical data and data on the health status, cigarette smoking, dietary habits, and other variables were collected by questionnaire and physical examination. Individuals from the town of Esperanza were exposed to drinking water As levels from two wells measured multiple times, with a combined mean of 43.3 ± 8.4 μg As/L. A comparable group of individuals from another town, Colonia Allende, were exposed to lower levels of As from one well of 5.5 ± 0.20 μg As/L. This well water was the sole source of drinking water for these subjects. Blood collection and processing were performed using similar methods to those described above for New Hampshire subjects.

First-morning-void urine samples were obtained in 100 mL polypropylene bottles and kept on ice. Within 6 hr, cooled samples were taken to the Institute Technologic of Sonora and kept frozen at −40°C. The accumulated samples were then shipped on dry ice to the University of Arizona, where the samples were stored at −80°C until the analysis of total As, and As species was performed as described previously (Meza et al. 2005). The detection limits were 0.42–1.08 μg/L for As compounds.

### Cell line

We used Jurkat lymphoblast cells as a controlled *in vitro* system to evaluate the effects of As on DNA damage and repair. Cells were grown in suspension in RPMI medium containing l -glutamine with 10% fetal bovine serum (Atlanta Biologicals, Norcross, GA, USA) and 1% penicillin-streptomycin (Mediatech Inc., Herndon, VA, USA). Cells were exposed to 0.01–10 μM sodium arsenite (Sigma, St. Louis, MO, USA), which is equivalent to 0.75–750 μg/L, for a period of 24 hr before harvesting and RNA isolation for gene expression analysis. Cells were exposed in culture to 0 or 1 μM (equivalent to 75 μg/L) As as sodium arsenite for 24 hr before the comet assay was performed, as described below.

### Gene expression analysis

RNA was harvested using Trizol reagent (Gibco/BRL Life Technologies, Gaithersburg, MD, USA) followed by DNase digestion using DNAfree (Ambion Inc., Austin, TX, USA) according to the manufacturer’s instructions and quantitated by spectrophotometric absorbance at 260 nm. We performed real-time reverse-transcription polymerase chain reaction (RT-PCR) using gene-specific primers and reagents (Applied Biosystems, Foster City, CA, USA) and the ABI PRISM sequence detection system and software (Applied Biosystems). Briefly, total RNA (0.5 μg) was reverse transcribed using 100 U M-MLV (Maloney murine leukemia virus) reverse transcriptase in a mixture with oligo-dT and dNTPs (deoxyribonucleotide triphosphates) according to the instructions provided with the Qiagen Omniscript kit (Qiagen, Valencia, CA, USA). Samples were reverse transcribed in a PTC-100 thermocycler (MJ Research Inc., Watertown, MA, USA) for 60 min at 44°C, and the reaction was terminated by heating to 95°C for 10 min. Expression of *ERCC1* [excision repair cross-complementing rodent repair deficiency, complementation group 1; GenBank gene ID 2067 (GenBank 2006)] was assessed by real-time PCR using 10 ng total RNA, 400 nM primers, 200 nM probe, and TaqMan Universal PCR Master Mix (Applied Biosystems). The sequence for the ERCC1 primer probe set is as follows: forward, CAGGACTTCGTCTCCCGGT; probe, TCTGGAACAGCTCATCGCCGCA; reverse, GCATAAGGCCAGATCTTCT-CTTG. Relative quantitation was performed using a standard curve consisting of serial dilutions of pooled sample cDNA from the same source as the test RNA with each plate. Relative expression levels of each gene were normalized to 18s rRNA or GAPDH (Applied Biosystems).

### Protein levels

We assessed the level of ERCC1 protein by immunoblotting using sodium dodecyl sulfate–polyacrylamide gel electrophoresis (SDS-PAGE) to resolve proteins from whole-cell lysates. Lymphocytes were rinsed with ice-cold stop buffer (10 mM Tris-HCl, pH 7.4, 10 mM EDTA, 5 mM EGTA, 100 mM NaF, 200 mM sucrose, 100 μM Na-orthovanadate, 5 mM pyrophosphate, 4 μg/mL leupeptin, 4 μg/mL soybean trypsin inhibitor, 1 mM benzamidine, 20 μM calpain inhibitor 1, 100 mU/mL aprotinin, and 100 μM phenylmethyl-sulfonylfluoride). The stop buffer was then replaced with a minimal volume of 2× SDS-PAGE buffer (62.5 mM Tris-HCl, pH 6.8, 10% glycerol, 2% SDS, 5% β-mercap-toethanol, 0.05% wt/vol bromophenol blue). The lysates were boiled for 5 min and clarified by centrifugation at 13,000 rpm for 10 min. Equal amounts of cell lysate were resolved by electrophoresis on 8–12% SDS-polyacrylamide gels. Electrophoresis was performed at constant voltage (200 V), and then the resolved proteins were transferred from the polyacrylamide gel to polyvinylidene difluoride membrane (PVDF; Immobilon-P; Millipore, Bedford, MA, USA) by semidry transfer (Hoeffer Semiphor, San Francisco, CA, USA) for 1 hr at constant current (32 mA/minigel) using transfer buffer (25 mM Tris, 192 mM glycine, 20% vol/vol methanol, 0.01% SDS). To eliminate non-specific interactions of antibodies with the membrane, the PVDF membrane was blocked with TTBS (10 mM Tris-HCl, pH 8.0, 150 mM NaCl, 0.05% Tween-20) containing 5% milk (wt/vol) for 1 hr at room temperature or overnight at 4°C. The membrane was incubated with the primary ERCC1 antibody (Neomarkers; Lab Vision Co., Fremont, CA, USA) diluted 1:200 in TTBS overnight at 4°C. The membrane was washed three times with TTBS and incubated with horseradish peroxidase–linked sheep anti-mouse IgG (1:2,000 in TTBS) (Amersham Pharmacia Biotech, Piscataway, NJ, USA) for 0.5–1 hr at room temperature. After three washes with TTBS, protein bands were visualized by enhanced chemiluminescence using the Renaissance system (NEN Life Sciences, Boston, MA, USA) and film (Lumi-Film; Roche Molecular Biochemicals, Indianapolis, IN, USA).

### DNA damage and repair assessment

The single-cell gel electrophoresis or comet assay is widely used to measure DNA damage and repair in primary human lymphocytes by measuring strand breaks and apurinic sites (Baltaci et al. 2002; De Silva et al. 2000; Rajaee-Behbahani et al. 2001; Schmezer et al. 2001). We divided the lymphocyte sample from each individual into parts to assess damage at several time points. We assessed baseline DNA damage levels as well as the capacity of the lymphocytes to repair damage induced by an *in vitro* challenge with 2-acetoxyacetyl-aminofluorene (2-AAAF; Midwest Research Institute, Kansas City, MO, USA), the reactive and genotoxic metabolite of 2-acetyl-aminofluorene. Alkaline-labile 2-AAAF adducts are primarily repaired through the nucleotide excision repair pathway (van Steeg 2001). Aliquots of lymphocytes were challenged for 2 hr *in vitro* with 4 μM 2-AAAF. A subset of 2-AAAF–challenged lymphocytes were incubated for an additional 4 hr to allow for DNA repair of the lesions. Comet analysis was performed using materials and protocols from Trevigen Inc. (Gaithersburg, MD, USA). Briefly, cells were mixed with agarose and spread over a warmed, precoated microscope slide. The agarose was allowed to solidify for 30 min at 4°C, followed by immersion in prechilled lysis solution for 45 min or overnight. Next, the slides were placed in freshly prepared alkaline solution, pH > 13, for 30 min at room temperature. The slides were then washed twice by immersion in 1× Tris-Borate-EDTA buffer for 5 min. Electrophoresis was carried out in alkaline buffer for 20 min at 1 V/cm (measured electrode to electrode) in the dark. Last, the slides were dipped into 70% ethanol for 5 min, allowed to dry completely, and stained with SYBR green (Trevigen). Image analysis of each cell was performed to quantify the length of the comet and the intensity of staining. All cells were analyzed using a fluorescence microscope coupled to the MD Biotech comet assay image analysis system (Morgantown, WV, USA). The Olive tail moment is a unitless measure of DNA damage that was calculated as described previously (Olive et al. 1990) using the quantity of migrated DNA multiplied by the distance between the comet head and the center of gravity of the DNA in the comet tail. The quantity of migrated DNA is the fraction of the DNA that has migrated from the head. The quantity of DNA is assessed as the DNA staining intensity subtracted from the background intensity. We scored the tail moment of all cells in a given well. Each point represents an average of 50 lymphocytes per individual (for human studies) or culture (for *in vitro* studies) from at least three to nine individuals or six cultures.

### Statistical analysis

We performed statistical analysis for gene expression and immunoblotting using analysis of variance with Newman-Keuls posttest, unpaired *t*-test, or linear regression procedures in GraphPad PRISM software (GraphPad Software Inc., San Diego, CA, USA). We considered *p*-values < 0.05 to be statistically significant. Statistical computations and graphics for comet analysis were performed using the S-Plus statistical package (version 6.2; Insightful Corporation, Seattle, WA, USA). We plotted the mean Olive tail moment with 95% confidence interval (CI) for each treatment group as a function of time. We performed an unpaired two-sided *t*-test to compare the low- and high-As groups at each time point. Linear regression was used to assess the slopes of the lines. Corresponding *p*-values are indicated.

## Results

Demographic characteristics of the study populations are shown in Table 1. A larger percentage of the subjects in Mexico were female. In addition, the Mexican population tended to be younger, with a mean age of 37 years, compared to a mean age of 64 years in New Hampshire subjects. Approximately one-third of each population consisted of smokers.

**Figure 1:**
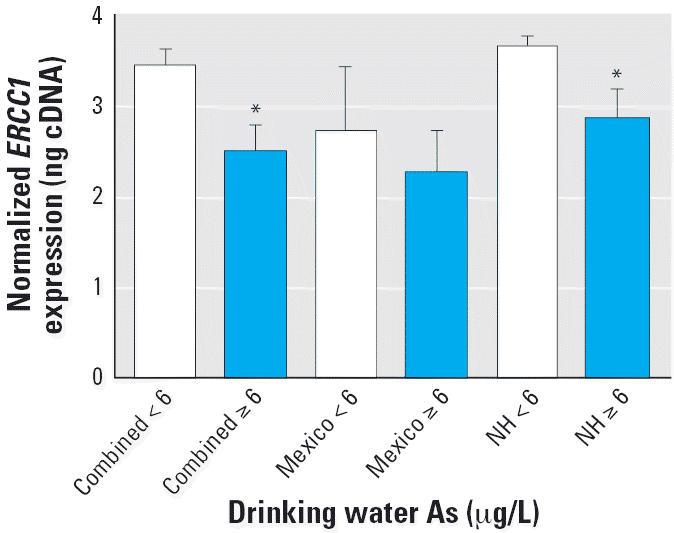
*ERCC1* gene expression (mean ± SE) by drinking water As level for individuals from the New Hampshire (NH) and Mexican populations and for both populations combined (*n* = 53). *ERCC1* levels were assessed by RT-PCR and normalized to 18s or GAPDH as described in “Materials and Methods.” *Statistically significant compared to the ≤ 5 μg/L group (*p* < 0.05).

As shown in Figure 1, analysis of both As-exposed populations combined indicated that individuals exposed to drinking water As concentrations ≥ 6 μg/L (*n* = 11) had lower *ERCC1* gene expression levels than those with < 6 μg/L As (*n* = 42; *p* < 0.05). The Mexican population alone had a lower *ERCC1* level among individuals exposed to As ≥ 6 μg/L, although this was not statistically significant. Likewise, a lower *ERCC1* level was observed in the New Hampshire individuals exposed to ≥ 6 μg/L As (statistically significant at *p* < 0.05).

We further assessed As exposure using available internal biomarkers of As level. However, different biomarkers were used in the two populations, which prevented pooling. Measurements included toenail As for the New Hampshire population and urinary As for the Mexican population. These markers correlate with drinking water As concentration (Karagas et al. 2000). Linear regression analysis of the New Hampshire population indicated an inverse association between toenail As levels and *ERCC1* expression (*r*^2^ = 0.4; *p* < 0.05). *ERCC1* expression decreased with increasing inorganic urinary As level [As(III) + As(V)] but not total urinary As (which may include organic As), although this was not statistically significant (*r*^2^ = 0.08; *p* = 0.3). In the New Hampshire population, we found no difference in *ERCC1* expression level according to cancer status (*p* = 0.8). For both populations, *ERCC1* expression did not significantly differ by smoking status, age, or sex (data not shown).

**Figure 2:**
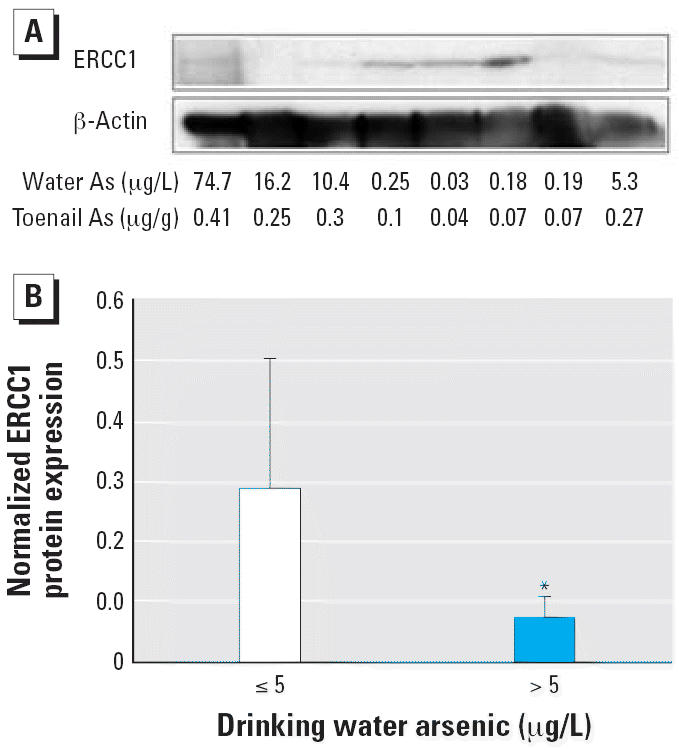
Association of drinking water As exposure > 5 μg/L with decreased ERCC1 protein levels in human lymphocytes from the New Hampshire population. (*A*) Immunoblot of protein extracts from human lymphocytes obtained from a subset of eight individuals assessed using antibody to ERCC1 or β -actin. (*B*) The ratio of band densities of ERCC1 to β-actin from the immunoblot shown in (*A*) graphed by drinking water As concentration; values shown are mean ± SD. *Statistically significant compared to the control group (*p* < 0.05).

We went on to investigate whether the decreased gene expression levels that we observed in lymphocytes of subjects exposed to As in New Hampshire translated to changes in protein levels. Immunoblots using an ERCC1 antibody indicated lower levels of ERCC1 protein among individuals exposed to drinking water As levels > 5 μg/L (*p* < 0.05), although there was some interindividual variation in expression (representative blot shown in Figure 2A, quantification shown in Figure 2B).

**Figure 3:**
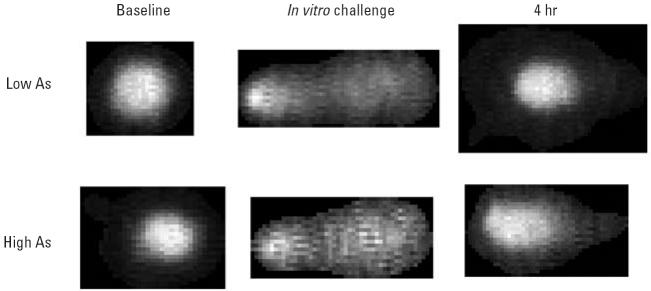
Comet assay on human lymphocytes obtained from New Hampshire subjects exposed *in vivo* to low or high drinking water As levels. Cells were analyzed at baseline, after *in vitro* challenge with 2-AAAF, and after a 4-hr repair period.

**Figure 4:**
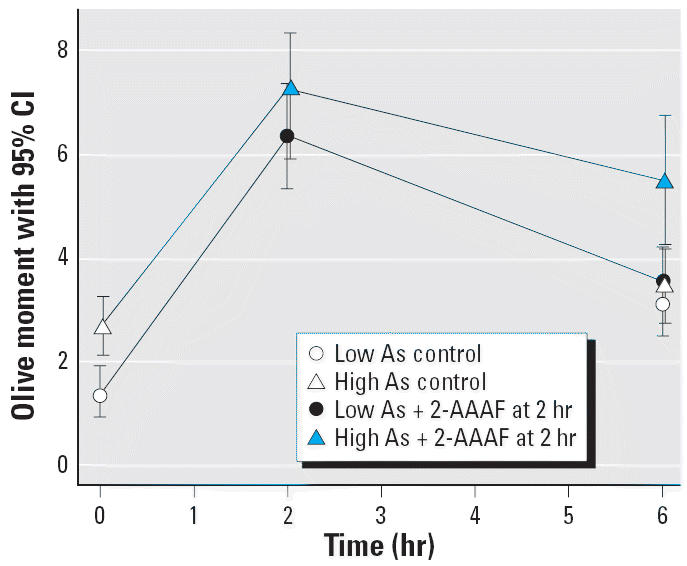
Comet assay results associated with *in vivo* As exposure in human lymphocytes from the New Hampshire population. Cells from 12 individual subjects were each divided into three subsets and analyzed immediately after harvest (time = 0 hr; *p* < 0.001), after a 2-hr *in vitro* challenge with 4 μM 2-AAAF (time = 2 hr; *p* = 0.248), and after a 4-hr repair period (time = 6 hr; *p* < 0.001). See “Materials and Methods” for details.

Additionally, we hypothesized that As exposure would be associated with correspondingly higher DNA damage levels and reduced DNA repair function. We analyzed human lymphocytes from a subset of New Hampshire residents exposed to low (< 0.7 μg/L) or high (≥ 13–93 μg/L) levels of drinking water As using the comet assay (Figure 3). We detected higher levels of DNA damage, as indicated by larger Olive tail moments, for lymphocytes analyzed at baseline from individuals exposed to high levels of drinking water As *in vivo* compared with those from lower level exposures (time 0 hr; *p* < 0.05). Analysis of these cells at 2 hr after 2-AAAF challenge demonstrated a dramatic increase in DNA damage but did not reveal any statistically significant differences in the amount of damage at 2 hr by As exposure status (*p* = 0.25). However, at the 6-hr time point, after the 4-hr repair period, significantly higher levels of DNA damage remained in lymphocytes from individuals exposed to high compared with low levels of As *in vivo* (*p* < 0.05) (Figure 4). Control lymphocytes that did not receive *in vitro* challenge showed similar levels of DNA damage at the 6-hr time point (Figure 4). The difference in slopes of the low- and high-As lines was not statistically significant.

**Figure 5:**
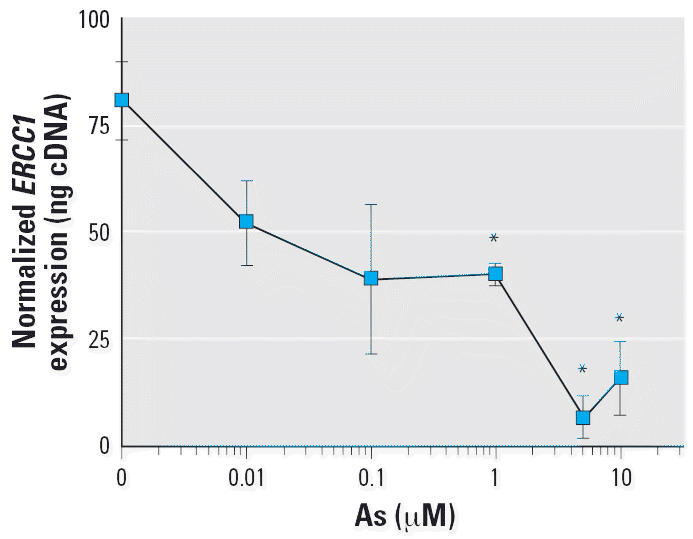
Effect of *in vitro* As exposure on *ERCC1* expression in a cultured Jurkat lymphoblast cell line. Cells were harvested after exposure to As (0.01–10 μM) for 24 hr. ERCC1 mRNA expression level was assessed by RT-PCR, quantitated using a standard curve using known amounts of cDNA, and normalized to 18s rRNA, as described in “Materials and Methods.” Values shown are mean ± SD. *Statistically significant compared to the control group (*p* < 0.05).

To further confirm the hypothesis that As exposure inhibits *ERCC1* expression, we repeated these experimental treatments using a human lymphoblast cell line. As shown in Figure 5, As suppressed *ERCC1* expression in the treated cells in a dose-responsive manner, beginning at the 0.1 μM (~ 7 μg/L) dose, with statistically significant decreases at ≥ 1 μM (*p* < 0.05) compared with unexposed controls.

**Figure 6:**
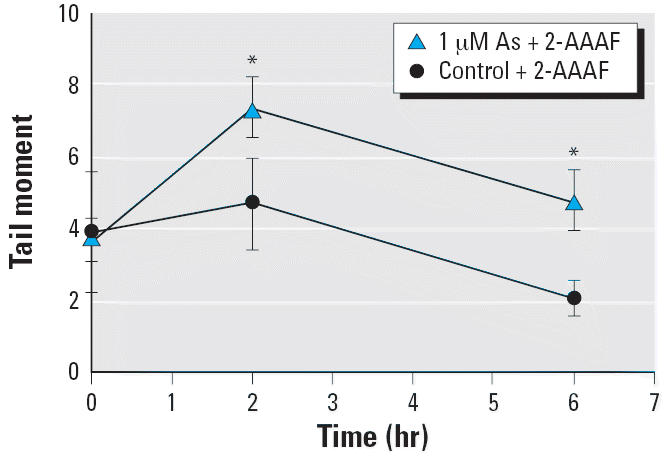
Effect of *in vitro* As exposure on DNA damage and repair function in a cultured Jurkat lymphoblast cell line. Cultured cells were exposed *in vitro* to 0 or 1 μM As for 24 hr (*n* = 6 independent cultures). Cells from each culture were analyzed by single-cell gel electrophoresis immediately after harvest (time = 0 hr), after a 2-hr *in vitro* challenge with 4 μM 2-AAAF (time = 2 hr), and after a 4-hr repair period (time = 6 hr). See “Materials and Methods” for details. Values represent mean ± 95% confidence interval. *Statistically significant compared to the control group (*p* < 0.05).

We further investigated the hypothesis that As exposure *in vitro* would decrease DNA repair function using the lymphoblast cell line. Arsenic-exposed and -unexposed cells had similar levels of baseline DNA damage at time 0 hr (Figure 6). Arsenic-exposed cells challenged with 2-AAAF for 2 hr showed significantly higher levels of DNA damage than did 2-AAAF–challenged cells that had not been exposed to As (time = 2 hr; *p* < 0.05; Figure 6). DNA damage for both As-exposed and -unexposed, 2-AAAF–challenged cells decreased during the 4-hr repair period (Figure 6). Nevertheless, DNA damage for As-exposed cells remained significantly higher than the nonexposed cells at the 6-hr time point (*p* < 0.05; Figure 6).

## Discussion

Elucidating the mechanism of As carcinogenicity has been challenging, in part due to the dose, time, and species specificity of its biologic effects (Barchowsky et al. 1999; WHO 2001). Our early study (Andrew et al. 2003a) supported previous *in vitro* work showing disruption of DNA repair gene expression by As. In the present study, we extended our findings to two different human populations at the gene expression, protein, and DNA repair functional levels. Thus, our data provide both human *in vivo* and *in vitro* support for the hypothesis that As inhibits DNA repair processes (ATSDR 1999; Hartwig 1998) and that this has the potential to affect subsequent exposure to other genotoxic and mutagenic agents.

The effects of As on DNA damage and repair have been evaluated almost exclusively in experimental systems. Previous *in vitro* studies demonstrated that As specifically interferes with the repair of DNA photolesions (Yang et al. 1992) and cross-linking agents (Yager and Wiencke 1993). In another study using human fibroblasts, Hartwig et al. (1997) found that low (2.5 μM) concentrations of arsenite reduced nucleotide excision repair efficiency, and incision frequency in particular, after UVR exposure. Results of additional studies in cultured human fibroblasts indicated that As exposure reduced DNA repair capacity and specifically inhibited the repair of UV-induced pyrimidine dimer-related DNA damage in lymphoblastoid cells as measured by the comet assay (Curnow et al. 2001; Danaee et al. 2004; Yager and Wiencke 1993).

Differences in gene expression results between these *in vitro* studies may be explained by differences in As dose because the effects of As are highly dose dependent. In the present study, treatment of lymphocytes with > 1 μM sodium arsenite *in vitro* decreased *ERCC1* gene expression. The circulating levels of As achieved in mice after intraperitoneally administering 0.625 nM As/kg body weight are approximately equivalent to the 5 μM *in vitro* dose and do not cause overt signs of toxicity (Soucy et al. 2003). In contrast, acutely toxic doses of As induced stress-response-pathway genes as well as *ERCC1* gene expression in the livers of mice injected with 100–300 μM As/kg body weight (Liu et al. 2001). Low concentrations of As induce cell proliferation, angiogenesis, hormone signaling, and nuclear factor κB–dependent transcription and do not appear to activate mitogen-activated protein kinase (MAPK) signaling or other stress response pathways. In contrast, high doses of As induce apoptosis and activate MAPKs, extracellular signal-regulated kinase (ERK), and p-38, as well as stress-mimetic and heat-shock–mimetic responses, inhibition of proliferation, and apoptosis (Barchowsky et al. 1999). Although decreased expression of ERCC1 may be partly responsible for the decreased DNA repair function associated with As exposure, we recognize that other pathway members may be involved, and future investigation will be needed to elucidate all factors involved. Because other environmental and genetic factors can influence DNA repair, we would not expect complete concordance between As exposure and expression or function on an individual level. Nevertheless, our *in vitro* studies demonstrate the effects of As within a controlled experimental system. Further work is needed to identify genotypes that modify the influence of As exposure on DNA repair.

In a human population, we previously found that drinking water As exposure at levels between 5 and 75 μg/L was associated with decreased mRNA expression of nucleotide excision repair pathway genes in lymphocytes from exposed individuals (Andrew et al. 2003a). Based on those preliminary results, we then followed up with the present study, which uses a larger human population in New Hampshire. In addition to enlarging the sample size, examination of a second population in Mexico exposed to moderate levels of As, supported our New Hampshire population results. Although we observed decreased *ERCC1* expression in both populations, the difference was not statistically significant in the Mexican population. The New Hampshire study had more extreme levels of exposure (Mexico, 5.5–43 μg/L; New Hampshire, 0.007–75 μg/L), but more likely the Mexico study had a smaller sample size and lacked statistical power. To our knowledge, no other studies have evaluated the association between As exposure and DNA repair gene expression or protein levels in human populations, particularly at As levels that are in the range that is routinely found in the United States.

In addition, our comet analysis provides functional DNA repair data in an As-exposed human population. These data support previous observations of decreased DNA repair capacity after As exposure *in vitro* (Hartwig 1998). Arsenite has been shown to inhibit DNA repair and act as a cogenotoxin for the direct-acting alkylating agent methyl methane-sulfonate, the indirect-acting PAH BaP, and UV-induced pyrimidine dimers in white blood cells and fibroblasts (Curnow et al. 2001; Danaee et al. 2004; Hartmann and Speit 1996; Okui and Fujiwara 1986). As observed by Danaee et al. (2004) in a previous study, our *in vitro* As exposure appeared to inhibit the fast component of DNA repair because the difference is observable after challenge (Figure 6, time = 2 hr). This difference in DNA migration remained significantly higher in the As-exposed group after the repair period (Figure 6, time = 6 hr). Thus, our study and others consistently report that As exacerbates DNA damage induced by other mutagens.

Whether inorganic As can directly induce DNA damage by itself is more controversial, and previous studies of DNA damage and mutagenesis by physiologic levels of inorganic As have been inconsistent (Mass et al. 2001; Schwerdtle et al. 2003; Sordo et al. 2001; Yih and Lee 2000). Our *in vitro* comet data did not show an increase in DNA migration after 24 hr treatment with 1 μM As alone (Figure 6, time = 0 hr), but we did observe higher DNA damage levels at baseline in cells harvested from individuals exposed to drinking water As at levels ≥ 13 μg/L compared to those with low levels of As (< 1 μg/L). The basis for this increase remains to be determined; however, higher levels of DNA damage in these lymphocytes after 2-AAAF challenge, and the slower repair kinetics of this damage, suggest that the higher baseline levels may be a result of As-inhibited repair and exposure to other DNA-damaging agents.

In summary, our *in vitro* studies of As exposure and our novel work using *in vivo* As exposure in two human populations support the hypothesis that As exposure decreases DNA repair capacity. Further, our data demonstrate decreased expression of the nucleotide excision repair pathway member ERCC1 and decreased repair after 2-AAAF challenge. These results support the theory that As can act through a cocarcinogenic mechanism of action, exacerbating the genotoxicity and mutagenicity of other compounds.

## Figures and Tables

**Table 1:** Percentage of the New Hampshire and Mexican populations with the selected characteristics.

	New Hampshire (*n* = 37)	Mexico (*n* = 16)	Combined (*n* = 53)
Sex			
Male	73	44	64
Female	27	56	36
Age (years)			
≤ 50	8	81	30
> 50	92	19	70
Current smoking			
Yes	32	38	34
No	68	62	66
Water As (μg/L)			
≤ 6	89	56	79
> 6	11	44	21
